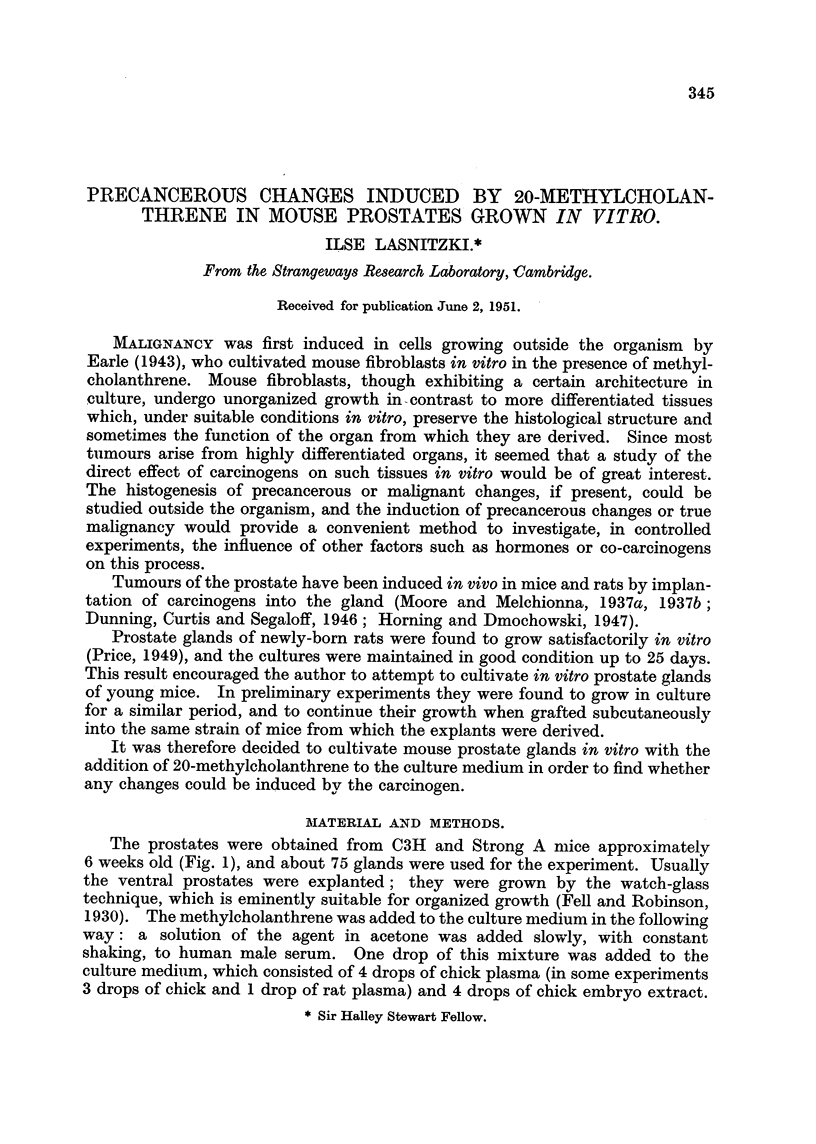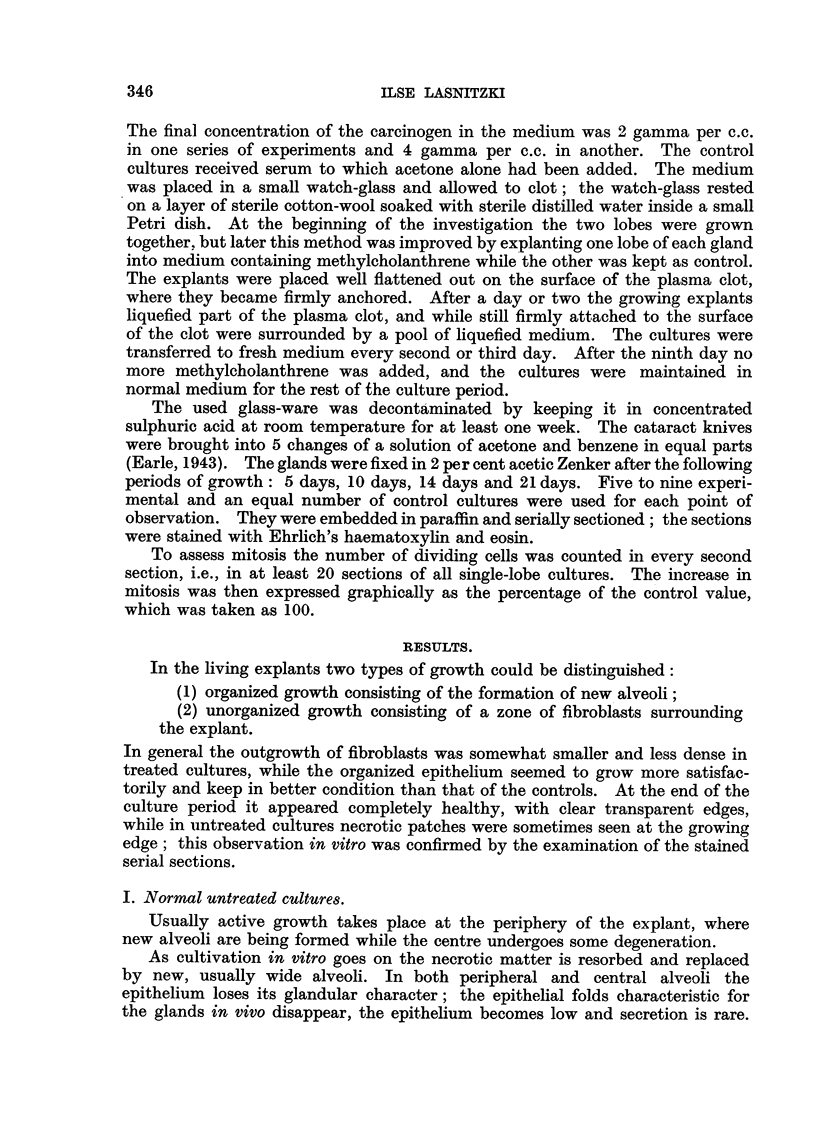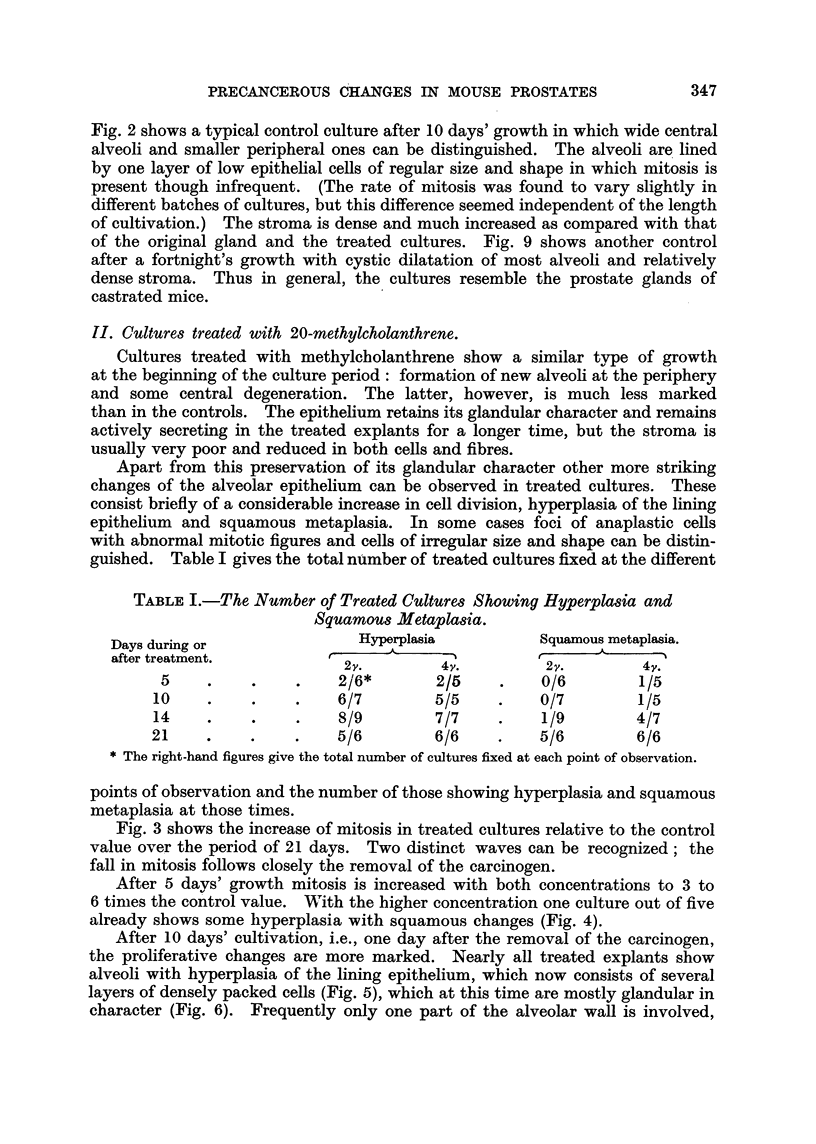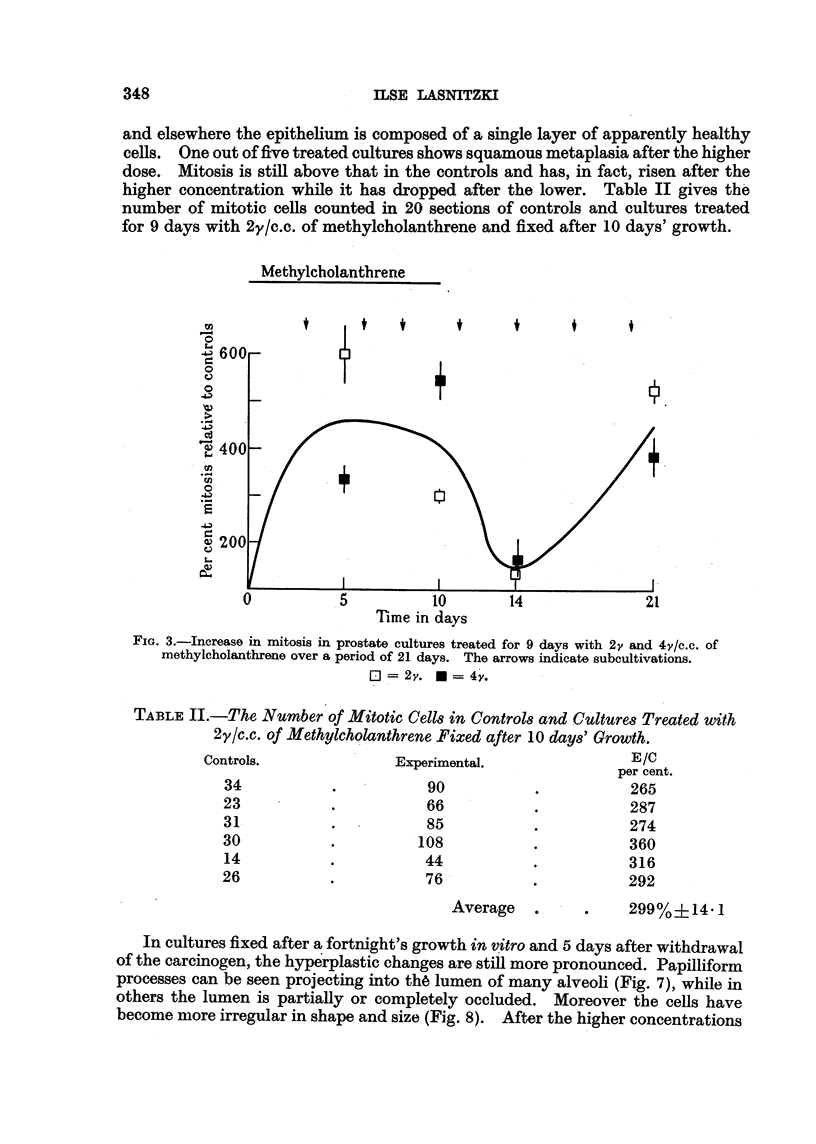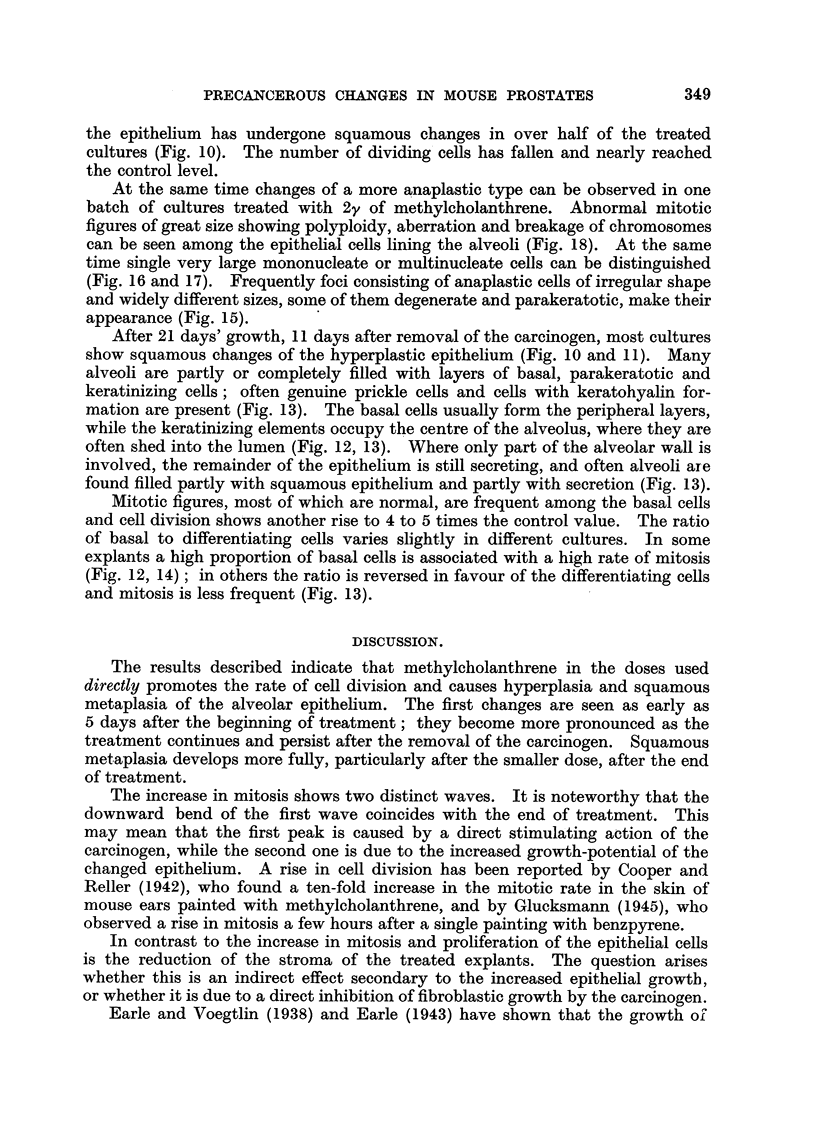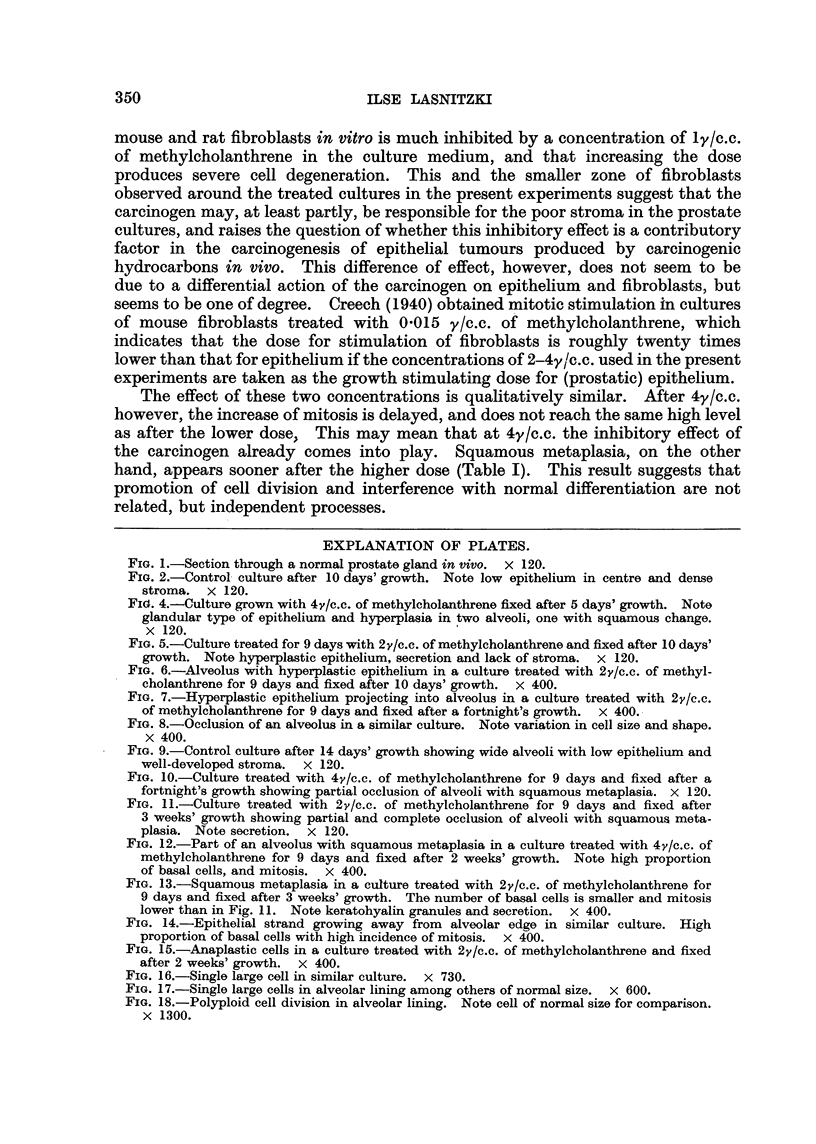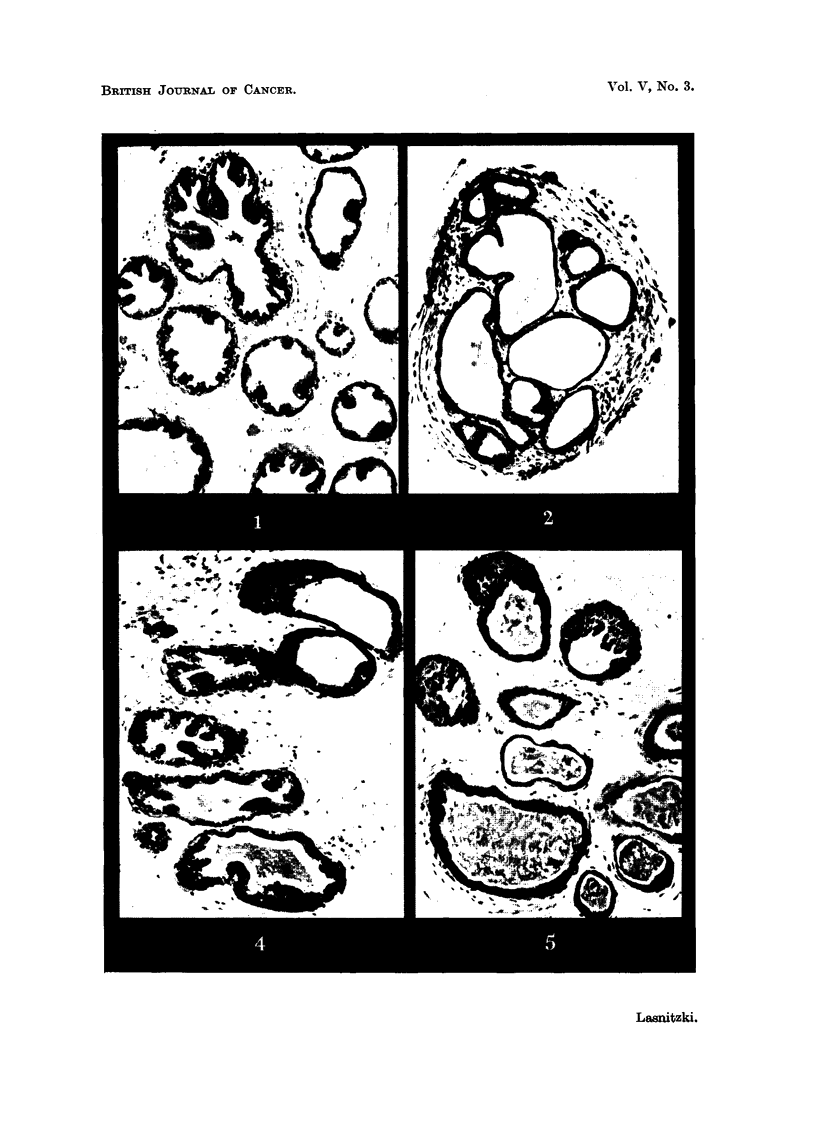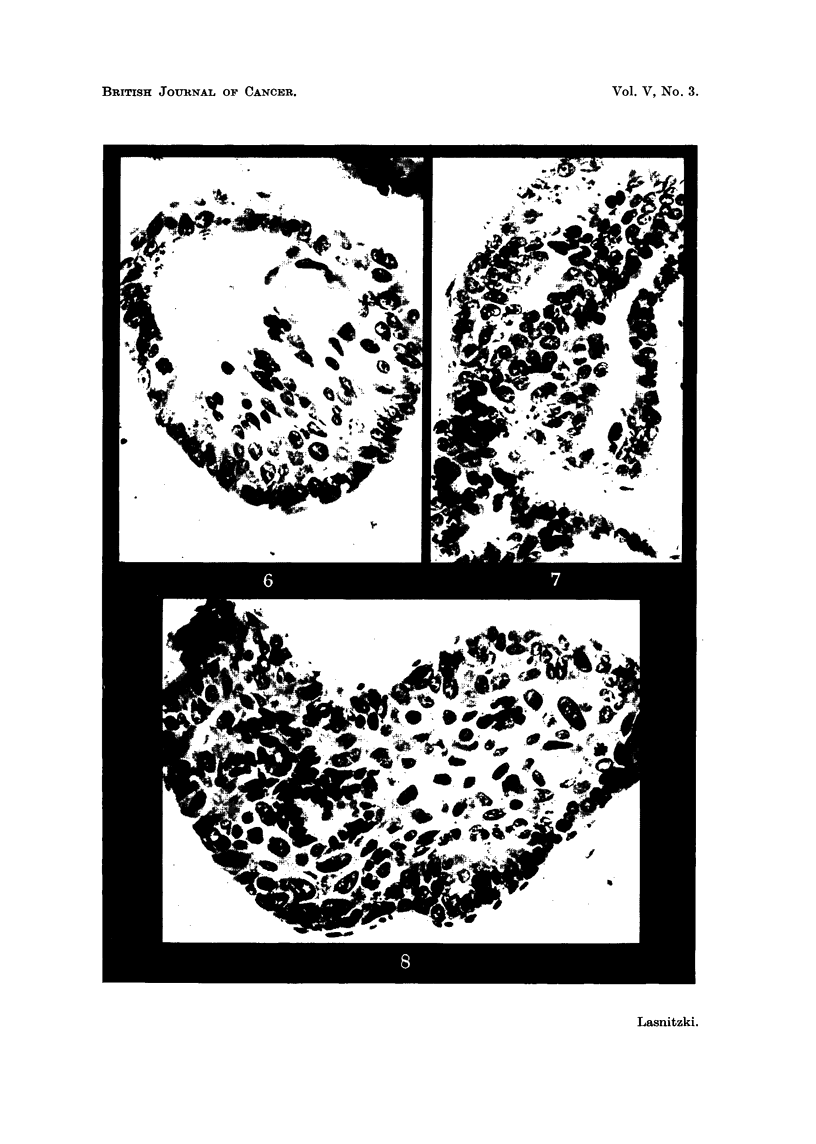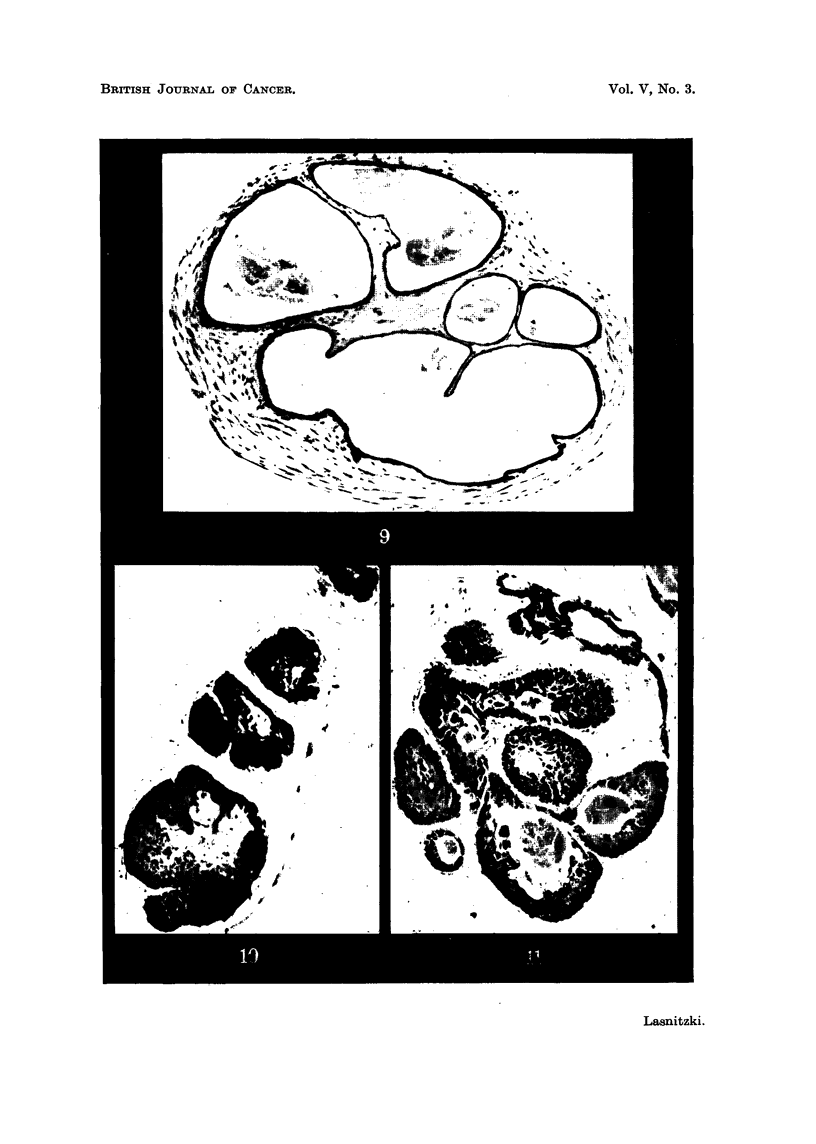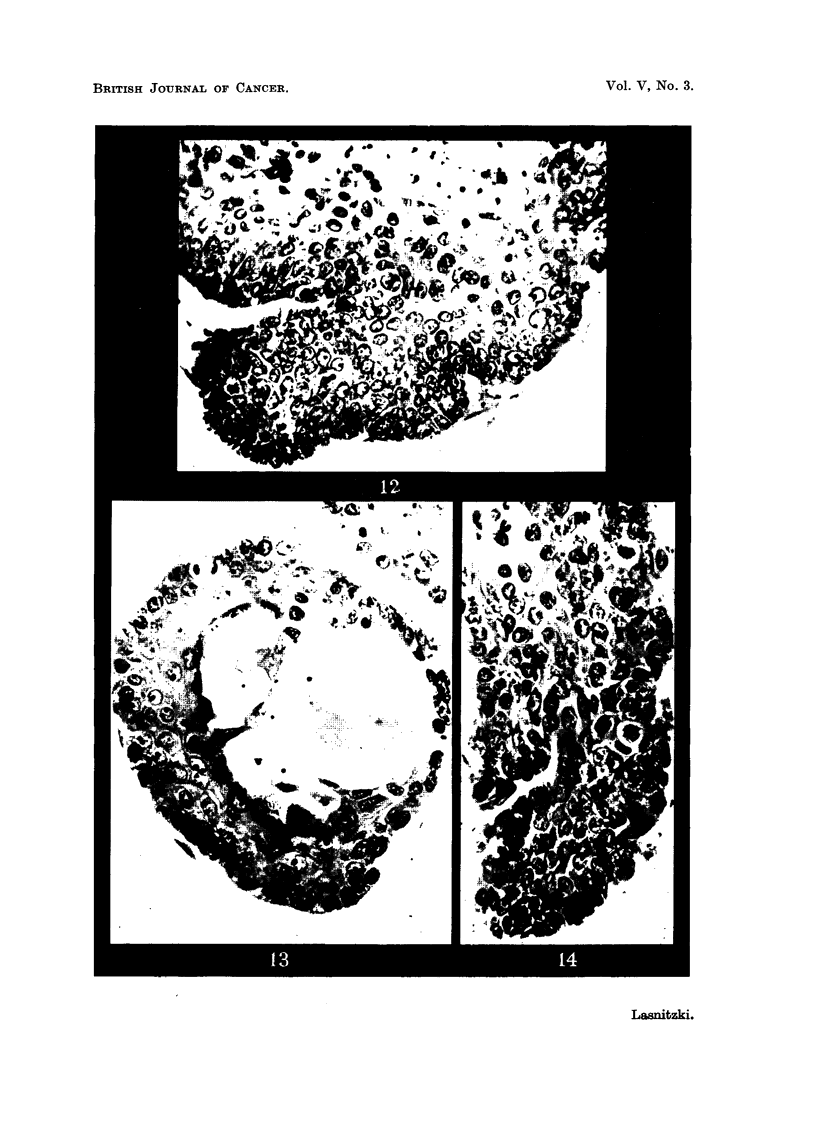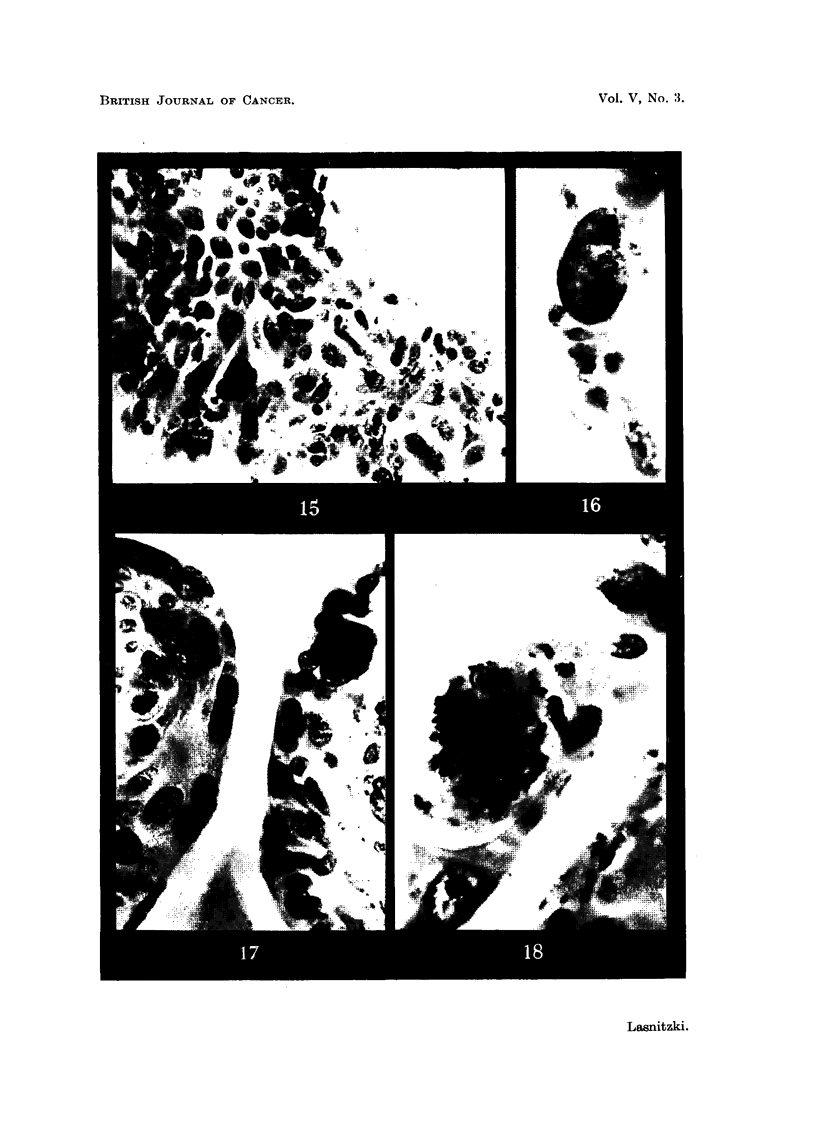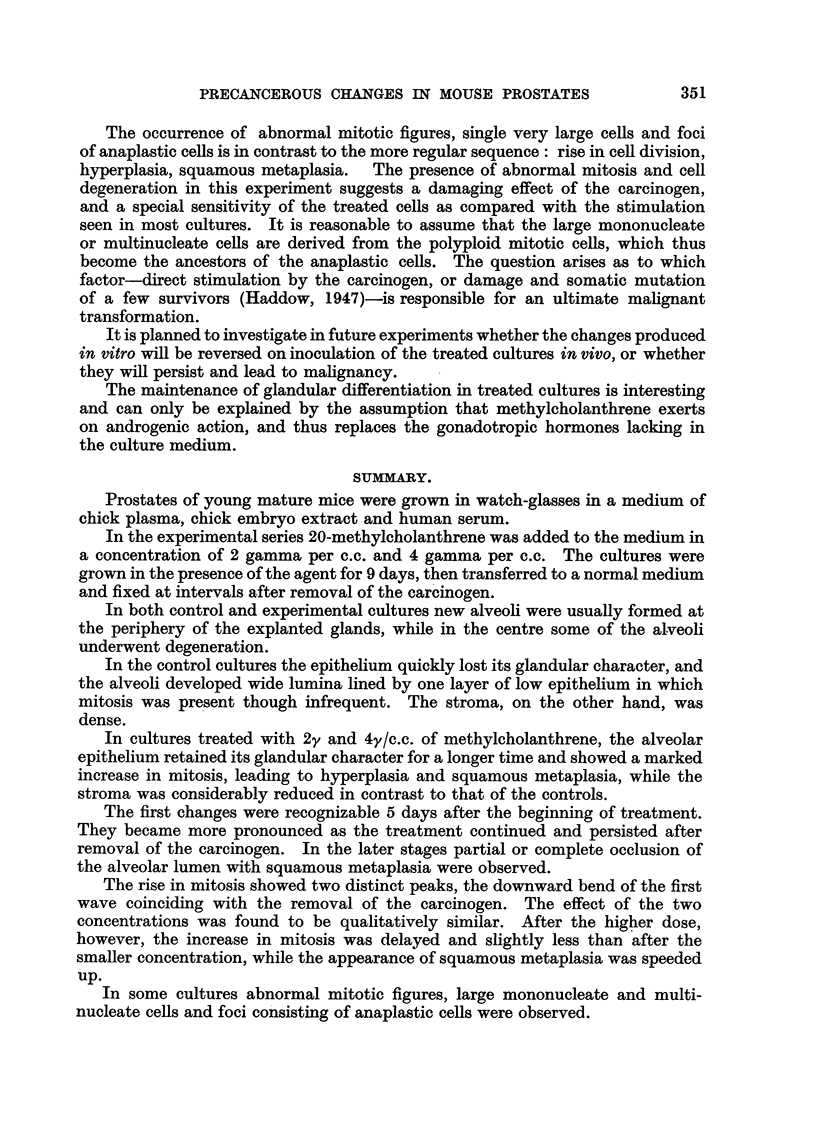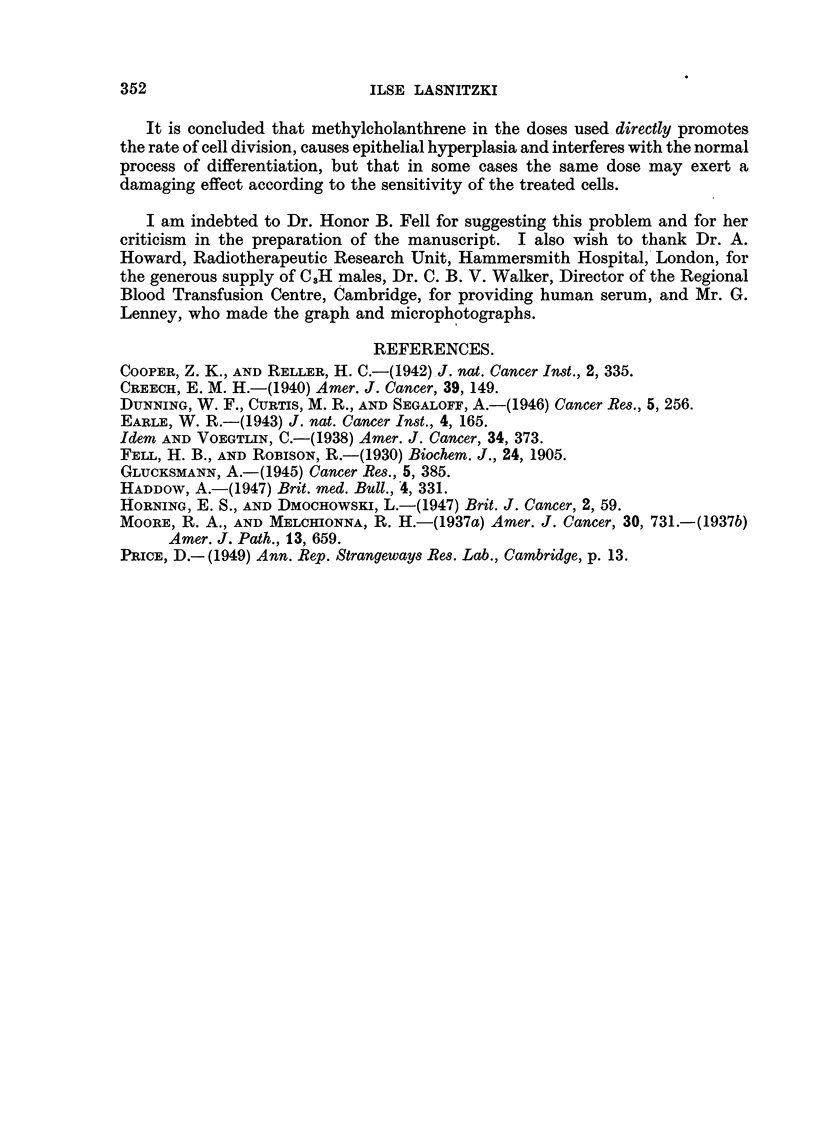# Precancerous Changes Induced by 20-Methylcholanthrene in Mouse Prostates Grown in vitro

**DOI:** 10.1038/bjc.1951.36

**Published:** 1951-09

**Authors:** Ilse Lasnitzki

## Abstract

**Images:**


### PRECANCEROUS CHANGES INDUCED BY 20-METHYLCHOLANTHRENE IN MOUSE PROSTATES GROWN *IN VITRO.*

ILSE LASNITZKI.*

*From the Strangeways Research Laboratory, Cambridge.*

Received for publication June 2, 1951.

MALIGNANCY was first induced in cells growing outside the organism by Earle (1943), who cultivated mouse fibroblasts *in vitro* in the presence of methylcholanthrene. Mouse fibroblasts, though exhibiting a certain architecture in culture, undergo unorganized growth in contrast to more differentiated tissues which, under suitable conditions *in vitro*, preserve the histological structure and sometimes the function of the organ from which they are derived. Since most tumours arise from highly differentiated organs, it seemed that a study of the direct effect of carcinogens on such tissues *in vitro* would be of great interest. The histogenesis of precancerous or malignant changes, if present, could be studied outside the organism, and the induction of precancerous changes or true malignancy would provide a convenient method to investigate, in controlled experiments, the influence of other factors such as hormones or co-carcinogens on this process.

Tumours of the prostate have been induced *in vivo* in mice and rats by implantation of carcinogens into the gland (Moore and Melchionna, 1937*a*, 1937*b* ; Dunning, Curtis and Segaloff, 1946 ; Horning and Dmochowski, 1947).

Prostate glands of newly-born rats were found to grow satisfactorily *in vitro* (Price, 1949), and the cultures were maintained in good condition up to 25 days. This result encouraged the author to attempt to cultivate *in vitro* prostate glands of young mice. In preliminary experiments they were found to grow in culture for a similar period, and to continue their growth when grafted subcutaneously into the same strain of mice from which the explants were derived.

It was therefore decided to cultivate mouse prostate glands *in vitro* with the addition of 20-methylcholanthrene to the culture medium in order to find whether any changes could be induced by the carcinogen.

### MATERIAL AND METHODS.

The prostates were obtained from C3H and Strong A mice approximately 6 weeks old (Fig. 1), and about 75 glands were used for the experiment. Usually the ventral prostates were explanted ; they were grown by the watch-glass technique, which is eminently suitable for organized growth (Fell and Robinson, 1930). The methylcholanthrene was added to the culture medium in the following way : a solution of the agent in acetone was added slowly, with constant shaking, to human male serum. One drop of this mixture was added to the culture medium, which consisted of 4 drops of chick plasma (in some experiments 3 drops of chick and 1 drop of rat plasma) and 4 drops of chick embryo extract.

* Sir Halley Stewart Fellow.

The final concentration of the carcinogen in the medium was 2 gamma per c.c. in one series of experiments and 4 gamma per c.c. in another. The control cultures received serum to which acetone alone had been added. The medium was placed in a small watch-glass and allowed to clot; the watch-glass rested on a layer of sterile cotton-wool soaked with sterile distilled water inside a small Petri dish. At the beginning of the investigation the two lobes were grown together, but later this method was improved by explanting one lobe of each gland into medium containing methylcholanthrene while the other was kept as control. The explants were placed well flattened out on the surface of the plasma clot, where they became firmly anchored. After a day or two the growing explants liquefied part of the plasma clot, and while still firmly attached to the surface of the clot were surrounded by a pool of liquefied medium. The cultures were transferred to fresh medium every second or third day. After the ninth day no more methylcholanthrene was added, and the cultures were maintained in normal medium for the rest of the culture period.

The used glass-ware was decontaminated by keeping it in concentrated sulphuric acid at room temperature for at least one week. The cataract knives were brought into 5 changes of a solution of acetone and benzene in equal parts (Earle, 1943). The glands were fixed in 2 per cent acetic Zenker after the following periods of growth: 5 days, 10 days, 14 days and 21 days. Five to nine experimental and an equal number of control cultures were used for each point of observation. They were embedded in paraffin and serially sectioned; the sections were stained with Ehrlich's haematoxylin and eosin.

To assess mitosis the number of dividing cells was counted in every second section, i.e., in at least 20 sections of all single-lobe cultures. The increase in mitosis was then expressed graphically as the percentage of the control value, which was taken as 100.

### RESULTS.

In the living explants two types of growth could be distinguished:

(1) organized growth consisting of the formation of new alveoli;

(2) unorganized growth consisting of a zone of fibroblasts surrounding the explant.

In general the outgrowth of fibroblasts was somewhat smaller and less dense in treated cultures, while the organized epithelium seemed to grow more satisfactorily and keep in better condition than that of the controls. At the end of the culture period it appeared completely healthy, with clear transparent edges, while in untreated cultures necrotic patches were sometimes seen at the growing edge; this observation *in vitro* was confirmed by the examination of the stained serial sections.

### I. *Normal untreated cultures.*

Usually active growth takes place at the periphery of the explant, where new alveoli are being formed while the centre undergoes some degeneration.

As cultivation *in vitro* goes on the necrotic matter is resorbed and replaced by new, usually wide alveoli. In both peripheral and central alveoli the epithelium loses its glandular character; the epithelial folds characteristic for the glands *in vivo* disappear, the epithelium becomes low and secretion is rare.

Fig. 2 shows a typical control culture after 10 days' growth in which wide central alveoli and smaller peripheral ones can be distinguished. The alveoli are lined by one layer of low epithelial cells of regular size and shape in which mitosis is present though infrequent. (The rate of mitosis was found to vary slightly in different batches of cultures, but this difference seemed independent of the length of cultivation.) The stroma is dense and much increased as compared with that of the original gland and the treated cultures. Fig. 9 shows another control after a fortnight's growth with cystic dilatation of most alveoli and relatively dense stroma. Thus in general, the cultures resemble the prostate glands of castrated mice.

*II. Cultures treated with* 20-*methylcholanthrene.*

Cultures treated with methylcholanthrene show a similar type of growth at the beginning of the culture period : formation of new alveoli at the periphery and some central degeneration. The latter, however, is much less marked than in the controls. The epithelium retains its glandular character and remains actively secreting in the treated explants for a longer time, but the stroma is usually very poor and reduced in both cells and fibres.

Apart from this preservation of its glandular character other more striking changes of the alveolar epithelium can be observed in treated cultures. These consist briefly of a considerable increase in cell division, hyperplasia of the lining epithelium and squamous metaplasia. In some cases foci of anaplastic cells with abnormal mitotic figures and cells of irregular size and shape can be distinguished. Table I gives the total number of treated cultures fixed at the different points of observation and the number of those showing hyperplasia and squamous metaplasia at those times.

TABLE I.—*The Number of Treated Cultures Showing Hyperplasia and Squamous Metaplasia.*

| Days during or after treatment. | Hyperplasia | | Squamous metaplasia. | |
|---|---|---|---|---|
| | 2γ. | 4γ. | 2γ. | 4γ. |
| 5 | 2/6* | 2/5 | 0/6 | 1/5 |
| 10 | 6/7 | 5/5 | 0/7 | 1/5 |
| 14 | 8/9 | 7/7 | 1/9 | 4/7 |
| 21 | 5/6 | 6/6 | 5/6 | 6/6 |

* The right-hand figures give the total number of cultures fixed at each point of observation.

Fig. 3 shows the increase of mitosis in treated cultures relative to the control value over the period of 21 days. Two distinct waves can be recognized ; the fall in mitosis follows closely the removal of the carcinogen.

After 5 days' growth mitosis is increased with both concentrations to 3 to 6 times the control value. With the higher concentration one culture out of five already shows some hyperplasia with squamous changes (Fig. 4).

After 10 days' cultivation, i.e., one day after the removal of the carcinogen, the proliferative changes are more marked. Nearly all treated explants show alveoli with hyperplasia of the lining epithelium, which now consists of several layers of densely packed cells (Fig. 5), which at this time are mostly glandular in character (Fig. 6). Frequently only one part of the alveolar wall is involved, and elsewhere the epithelium is composed of a single layer of apparently healthy cells. One out of five treated cultures shows squamous metaplasia after the higher dose. Mitosis is still above that in the controls and has, in fact, risen after the higher concentration while it has dropped after the lower. Table II gives the number of mitotic cells counted in 20 sections of controls and cultures treated for 9 days with 2γ/c.c. of methylcholanthrene and fixed after 10 days' growth.

Fig. 3.—Increase in mitosis in prostate cultures treated for 9 days with 2γ and 4γ/c.c. of methylcholanthrene over a period of 21 days. The arrows indicate subcultivations.
□ = 2γ. ■ = 4γ.

Table II.—*The Number of Mitotic Cells in Controls and Cultures Treated with 2γ/c.c. of Methylcholanthrene Fixed after* 10 *days' Growth.*

| Controls. | Experimental. | E/C per cent. |
|---|---|---|
| 34 | 90 | 265 |
| 23 | 66 | 287 |
| 31 | 85 | 274 |
| 30 | 108 | 360 |
| 14 | 44 | 316 |
| 26 | 76 | 292 |
| | Average | 299%±14·1 |

In cultures fixed after a fortnight's growth *in vitro* and 5 days after withdrawal of the carcinogen, the hyperplastic changes are still more pronounced. Papilliform processes can be seen projecting into the lumen of many alveoli (Fig. 7), while in others the lumen is partially or completely occluded. Moreover the cells have become more irregular in shape and size (Fig. 8). After the higher concentrations the epithelium has undergone squamous changes in over half of the treated cultures (Fig. 10). The number of dividing cells has fallen and nearly reached the control level.

At the same time changes of a more anaplastic type can be observed in one batch of cultures treated with $2\gamma$ of methylcholanthrene. Abnormal mitotic figures of great size showing polyploidy, aberration and breakage of chromosomes can be seen among the epithelial cells lining the alveoli (Fig. 18). At the same time single very large mononucleate or multinucleate cells can be distinguished (Fig. 16 and 17). Frequently foci consisting of anaplastic cells of irregular shape and widely different sizes, some of them degenerate and parakeratotic, make their appearance (Fig. 15).

After 21 days' growth, 11 days after removal of the carcinogen, most cultures show squamous changes of the hyperplastic epithelium (Fig. 10 and 11). Many alveoli are partly or completely filled with layers of basal, parakeratotic and keratinizing cells ; often genuine prickle cells and cells with keratohyalin formation are present (Fig. 13). The basal cells usually form the peripheral layers, while the keratinizing elements occupy the centre of the alveolus, where they are often shed into the lumen (Fig. 12, 13). Where only part of the alveolar wall is involved, the remainder of the epithelium is still secreting, and often alveoli are found filled partly with squamous epithelium and partly with secretion (Fig. 13).

Mitotic figures, most of which are normal, are frequent among the basal cells and cell division shows another rise to 4 to 5 times the control value. The ratio of basal to differentiating cells varies slightly in different cultures. In some explants a high proportion of basal cells is associated with a high rate of mitosis (Fig. 12, 14) ; in others the ratio is reversed in favour of the differentiating cells and mitosis is less frequent (Fig. 13).

### DISCUSSION.

The results described indicate that methylcholanthrene in the doses used *directly* promotes the rate of cell division and causes hyperplasia and squamous metaplasia of the alveolar epithelium. The first changes are seen as early as 5 days after the beginning of treatment ; they become more pronounced as the treatment continues and persist after the removal of the carcinogen. Squamous metaplasia develops more fully, particularly after the smaller dose, after the end of treatment.

The increase in mitosis shows two distinct waves. It is noteworthy that the downward bend of the first wave coincides with the end of treatment. This may mean that the first peak is caused by a direct stimulating action of the carcinogen, while the second one is due to the increased growth-potential of the changed epithelium. A rise in cell division has been reported by Cooper and Reller (1942), who found a ten-fold increase in the mitotic rate in the skin of mouse ears painted with methylcholanthrene, and by Glucksmann (1945), who observed a rise in mitosis a few hours after a single painting with benzpyrene.

In contrast to the increase in mitosis and proliferation of the epithelial cells is the reduction of the stroma of the treated explants. The question arises whether this is an indirect effect secondary to the increased epithelial growth, or whether it is due to a direct inhibition of fibroblastic growth by the carcinogen.

Earle and Voegtlin (1938) and Earle (1943) have shown that the growth of mouse and rat fibroblasts *in vitro* is much inhibited by a concentration of 1γ/c.c. of methylcholanthrene in the culture medium, and that increasing the dose produces severe cell degeneration. This and the smaller zone of fibroblasts observed around the treated cultures in the present experiments suggest that the carcinogen may, at least partly, be responsible for the poor stroma in the prostate cultures, and raises the question of whether this inhibitory effect is a contributory factor in the carcinogenesis of epithelial tumours produced by carcinogenic hydrocarbons *in vivo.* This difference of effect, however, does not seem to be due to a differential action of the carcinogen on epithelium and fibroblasts, but seems to be one of degree. Creech (1940) obtained mitotic stimulation in cultures of mouse fibroblasts treated with 0·015 γ/c.c. of methylcholanthrene, which indicates that the dose for stimulation of fibroblasts is roughly twenty times lower than that for epithelium if the concentrations of 2–4γ/c.c. used in the present experiments are taken as the growth stimulating dose for (prostatic) epithelium.

The effect of these two concentrations is qualitatively similar. After 4γ/c.c. however, the increase of mitosis is delayed, and does not reach the same high level as after the lower dose, This may mean that at 4γ/c.c. the inhibitory effect of the carcinogen already comes into play. Squamous metaplasia, on the other hand, appears sooner after the higher dose (Table I). This result suggests that promotion of cell division and interference with normal differentiation are not related, but independent processes.

---

### EXPLANATION OF PLATES.

FIG. 1.—Section through a normal prostate gland *in vivo*. × 120.

FIG. 2.—Control culture after 10 days' growth. Note low epithelium in centre and dense stroma. × 120.

FIG. 4.—Culture grown with 4γ/c.c. of methylcholanthrene fixed after 5 days' growth. Note glandular type of epithelium and hyperplasia in two alveoli, one with squamous change. × 120.

FIG. 5.—Culture treated for 9 days with 2γ/c.c. of methylcholanthrene and fixed after 10 days' growth. Note hyperplastic epithelium, secretion and lack of stroma. × 120.

FIG. 6.—Alveolus with hyperplastic epithelium in a culture treated with 2γ/c.c. of methylcholanthrene for 9 days and fixed after 10 days' growth. × 400.

FIG. 7.—Hyperplastic epithelium projecting into alveolus in a culture treated with 2γ/c.c. of methylcholanthrene for 9 days and fixed after a fortnight's growth. × 400.

FIG. 8.—Occlusion of an alveolus in a similar culture. Note variation in cell size and shape. × 400.

FIG. 9.—Control culture after 14 days' growth showing wide alveoli with low epithelium and well-developed stroma. × 120.

FIG. 10.—Culture treated with 4γ/c.c. of methylcholanthrene for 9 days and fixed after a fortnight's growth showing partial occlusion of alveoli with squamous metaplasia. × 120.

FIG. 11.—Culture treated with 2γ/c.c. of methylcholanthrene for 9 days and fixed after 3 weeks' growth showing partial and complete occlusion of alveoli with squamous metaplasia. Note secretion. × 120.

FIG. 12.—Part of an alveolus with squamous metaplasia in a culture treated with 4γ/c.c. of methylcholanthrene for 9 days and fixed after 2 weeks' growth. Note high proportion of basal cells, and mitosis. × 400.

FIG. 13.—Squamous metaplasia in a culture treated with 2γ/c.c. of methylcholanthrene for 9 days and fixed after 3 weeks' growth. The number of basal cells is smaller and mitosis lower than in Fig. 11. Note keratohyalin granules and secretion. × 400.

FIG. 14.—Epithelial strand growing away from alveolar edge in similar culture. High proportion of basal cells with high incidence of mitosis. × 400.

FIG. 15.—Anaplastic cells in a culture treated with 2γ/c.c. of methylcholanthrene and fixed after 2 weeks' growth. × 400.

FIG. 16.—Single large cell in similar culture. × 730.

FIG. 17.—Single large cells in alveolar lining among others of normal size. × 600.

FIG. 18.—Polyploid cell division in alveolar lining. Note cell of normal size for comparison. × 1300.

6

7

8

Lasnitzki.

9

10

11

12

13

14

Lasnitzki.

15

16

17

18

The occurrence of abnormal mitotic figures, single very large cells and foci of anaplastic cells is in contrast to the more regular sequence : rise in cell division, hyperplasia, squamous metaplasia. The presence of abnormal mitosis and cell degeneration in this experiment suggests a damaging effect of the carcinogen, and a special sensitivity of the treated cells as compared with the stimulation seen in most cultures. It is reasonable to assume that the large mononucleate or multinucleate cells are derived from the polyploid mitotic cells, which thus become the ancestors of the anaplastic cells. The question arises as to which factor—direct stimulation by the carcinogen, or damage and somatic mutation of a few survivors (Haddow, 1947)—is responsible for an ultimate malignant transformation.

It is planned to investigate in future experiments whether the changes produced *in vitro* will be reversed on inoculation of the treated cultures *in vivo*, or whether they will persist and lead to malignancy.

The maintenance of glandular differentiation in treated cultures is interesting and can only be explained by the assumption that methylcholanthrene exerts on androgenic action, and thus replaces the gonadotropic hormones lacking in the culture medium.

### SUMMARY.

Prostates of young mature mice were grown in watch-glasses in a medium of chick plasma, chick embryo extract and human serum.

In the experimental series 20-methylcholanthrene was added to the medium in a concentration of 2 gamma per c.c. and 4 gamma per c.c. The cultures were grown in the presence of the agent for 9 days, then transferred to a normal medium and fixed at intervals after removal of the carcinogen.

In both control and experimental cultures new alveoli were usually formed at the periphery of the explanted glands, while in the centre some of the alveoli underwent degeneration.

In the control cultures the epithelium quickly lost its glandular character, and the alveoli developed wide lumina lined by one layer of low epithelium in which mitosis was present though infrequent. The stroma, on the other hand, was dense.

In cultures treated with $2\gamma$ and $4\gamma$/c.c. of methylcholanthrene, the alveolar epithelium retained its glandular character for a longer time and showed a marked increase in mitosis, leading to hyperplasia and squamous metaplasia, while the stroma was considerably reduced in contrast to that of the controls.

The first changes were recognizable 5 days after the beginning of treatment. They became more pronounced as the treatment continued and persisted after removal of the carcinogen. In the later stages partial or complete occlusion of the alveolar lumen with squamous metaplasia were observed.

The rise in mitosis showed two distinct peaks, the downward bend of the first wave coinciding with the removal of the carcinogen. The effect of the two concentrations was found to be qualitatively similar. After the higher dose, however, the increase in mitosis was delayed and slightly less than after the smaller concentration, while the appearance of squamous metaplasia was speeded up.

In some cultures abnormal mitotic figures, large mononucleate and multinucleate cells and foci consisting of anaplastic cells were observed.

It is concluded that methylcholanthrene in the doses used *directly* promotes the rate of cell division, causes epithelial hyperplasia and interferes with the normal process of differentiation, but that in some cases the same dose may exert a damaging effect according to the sensitivity of the treated cells.

I am indebted to Dr. Honor B. Fell for suggesting this problem and for her criticism in the preparation of the manuscript. I also wish to thank Dr. A. Howard, Radiotherapeutic Research Unit, Hammersmith Hospital, London, for the generous supply of $C_3H$ males, Dr. C. B. V. Walker, Director of the Regional Blood Transfusion Centre, Cambridge, for providing human serum, and Mr. G. Lenney, who made the graph and microphotographs.

### REFERENCES.

Cooper, Z. K., and Reller, H. C.—(1942) *J. nat. Cancer Inst.*, **2**, 335.
Creech, E. M. H.—(1940) *Amer. J. Cancer*, **39**, 149.
Dunning, W. F., Curtis, M. R., and Segaloff, A.—(1946) *Cancer Res.*, **5**, 256.
Earle, W. R.—(1943) *J. nat. Cancer Inst.*, **4**, 165.
*Idem* and Voegtlin, C.—(1938) *Amer. J. Cancer*, **34**, 373.
Fell, H. B., and Robison, R.—(1930) *Biochem. J.*, **24**, 1905.
Glucksmann, A.—(1945) *Cancer Res.*, **5**, 385.
Haddow, A.—(1947) *Brit. med. Bull.*, **4**, 331.
Horning, E. S., and Dmochowski, L.—(1947) *Brit. J. Cancer*, **2**, 59.
Moore, R. A., and Melchionna, R. H.—(1937*a*) *Amer. J. Cancer*, **30**, 731.—(1937*b*) *Amer. J. Path.*, **13**, 659.
Price, D.—(1949) *Ann. Rep. Strangeways Res. Lab., Cambridge*, p. 13.